# Supplementary material for: IL-33 induces thymic involution-associated naive T cell aging and impairs host control of severe infection
Source: Nat Commun. 2022 Nov 12;13:6881. doi: 10.1038/s41467-022-34660-4 (PMC9653498; doi:10.1038/s41467-022-34660-4)
Supplement: Supplementary file 1 — Supplementary Information [file 41467_2022_34660_MOESM1_ESM.pdf]

Supplementary Information

Supplementary Figures 1-7 and Legends

IL-33 induces thymic involution-associated naive T cell aging and impairs host control of severe infection

Lei Xu [1, 2, 3, 4, 5, 6, #], Chuan Wei [1, 2, 3, 4, #], Ying Chen [1, 2, 3, 4], Yue Wu [1, 2, 3, 4], Xiaoli Shou [1, 2, 3, 4], Wenjie Chen [1, 2, 3, 4], Di Lu [1, 2, 3, 4], Haoran Sun [1, 2, 3, 4], Wei Li [6], Beibei Yu [1, 2, 3, 4], Xiaowei Wang [7], Xiaojun Zhang [8], Yanxiong Yu [1, 2, 3, 4], Zhigang Lei [1, 2, 3, 4], Rui Tang [1, 2, 3, 4], Jifeng Zhu [1, 2, 3, 4], Yalin Li [1, 2, 3, 4], Linrong Lu [9], Hong Zhou [10], Sha Zhou [1, 2, 3, 4, *], Chuan Su [1, 2, 3, 4, *], Xiaojun Chen [1, 2, 3, 4, *]

[1]Jiangsu Key Laboratory of Pathogen Biology, Nanjing Medical University, Nanjing, Jiangsu 211166, P. R. China.

[2]State Key Lab of Reproductive Medicine, Nanjing Medical University, Nanjing, Jiangsu 211166, P. R. China.

[3]Department of Pathogen Biology and Immunology, Nanjing Medical University, Nanjing, Jiangsu 211166, P. R. China.

[4]Center for Global Health, Nanjing Medical University, Nanjing, Jiangsu 211166, P. R. China.

[5]Department of Respiratory, Nanjing First Hospital, Nanjing Medical University, Nanjing, Jiangsu 210006, P. R. China.

[6]Department of Clinical Laboratory, Nanjing First Hospital, Nanjing Medical University, Nanjing, Jiangsu 210006, P. R. China.

[7]Department of Blood Transfusion, Children's Hospital of Nanjing Medical University, Nanjing, Jiangsu 210008, P. R. China.

[8]Imaging Center, Children’s Hospital of Nanjing Medical University, Nanjing, Jiangsu 210008, P. R. China.

[9]Institute of Immunology, School of Medicine, Zhejiang University, Hangzhou 310058, P. R. China.

[10]Department of Cell Biology, School of Life Sciences, Anhui Medical University, Hefei 230032, P. R. China.

[#]These individuals contributed equally.

[*]Correspondence and requests for materials should be addressed to X.C. (email: chenxiaojun@njmu.edu.cn), C.S. (email: chuansu@njmu.edu.cn), or S.Z. (shazhou@ njmu.edu.cn).

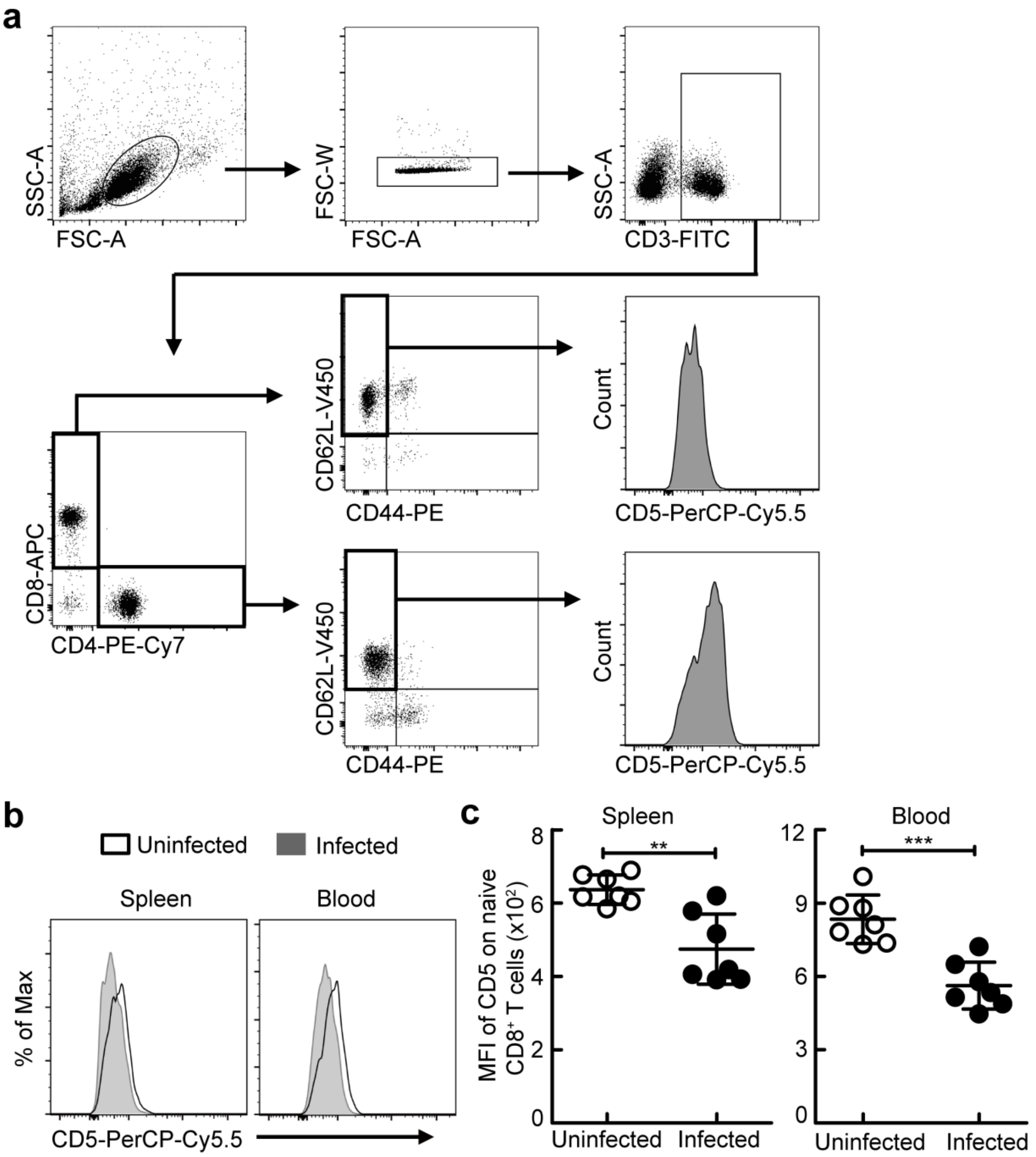

Fig. S1. Schistosome infection decreases the expression of CD5 in naïve CD8$^+$ T cells. (a) Gating strategy of CD5 expression on naïve CD4$^+$ T cells or naïve CD8$^+$ T cells; (**b, c**) Samples were obtained from uninfected mice or mice 8 weeks after schistosome infection. Representative and quantified flow cytometry of CD5 MFI on naive CD8$^+$ T cells from the spleen or peripheral blood (n = 7 mice, pool of two independent experiments). P = 0.0014 (Spleen), P = 0.0002 (Blood), Unpaired two-tailed Student's t-test. All data are shown as the mean ± s.d. **P < 0.01, ***P < 0.001. Source data are provided as a Source Data file.

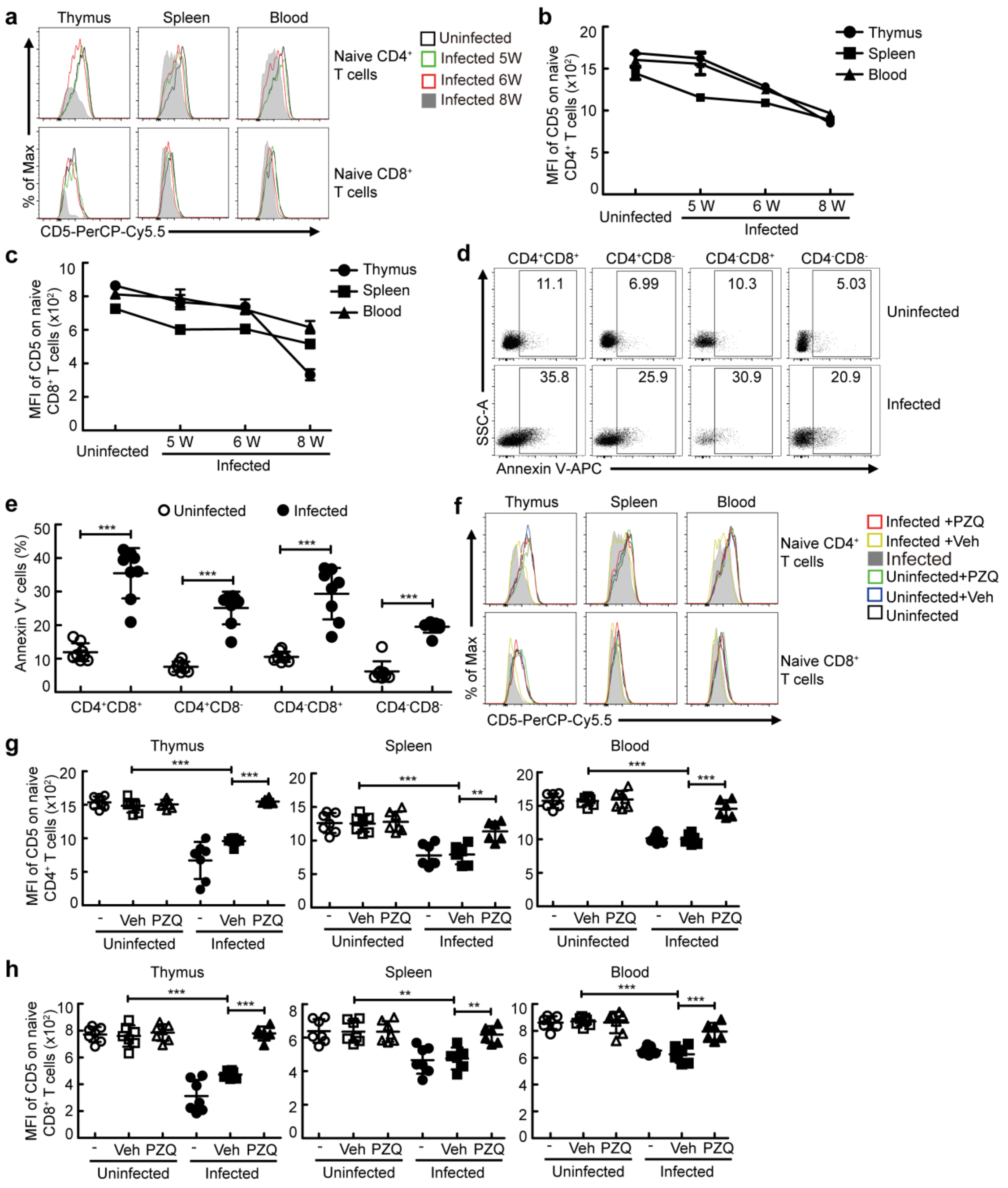

Fig. S2. T cell aging is linked to thymic involution during schistosome infection. (**a-c**) Cells were from uninfected mice or mice 5, 6, or 8 weeks after schistosome infection. Representative and quantified flow cytometry of CD5 MFI on naïve CD4$^+$ T cells or naïve CD8$^+$ T cells from the thymus, spleen, or peripheral blood (Infected 8 W, n = 3 mice; other groups, n = 4 mice). (**d, e**) Samples were obtained from uninfected mice or mice 8 weeks after schistosome infection. Representative and quantified flow cytometry of annexin V$^+$ cells gated on CD4$^+$CD8$^+$DP, CD4SP, CD8SP, or CD4$^-$CD8$^-$ DN thymocytes in the thymus (n = 8 mice, pool of two independent experiments). P < 0.0001 for all thymocyte subsets, One-way

ANOVA with Tukey's multiple comparisons. (**f-h**) Mice were treated with PZQ at week 8 after infection and sacrificed 7 weeks after PZQ treatment. Representative and quantified flow cytometry of CD5 MFI on naive $CD4^+$ T cells or naïve $CD8^+$ T cells from the thymus, spleen, or peripheral blood (Infected+Veh or Infected+PZQ, n = 6 mice; other groups, n = 7 mice; pool of two independent experiments). $CD4^+$ T cells: Infected+Veh versus Uninfected+Veh, $P < 0.0001$ (Thymus), $P < 0.0001$ (Spleen), $P < 0.0001$ (Blood); Infected+PZQ versus Infected+Veh, $P < 0.0001$ (Thymus), $P = 0.0035$ (Spleen), $P < 0.0001$ (Blood). $CD8^+$ T cells: Infected+Veh versus Uninfected+Veh, $P < 0.0001$ (Thymus), $P = 0.0017$ (Spleen), $P < 0.0001$ (Blood); Infected+PZQ versus Infected+Veh, $P < 0.0001$ (Thymus), $P = 0.0094$ (Spleen), $P < 0.0001$ (Blood). One-way ANOVA with Tukey's multiple comparisons. All data are shown as the mean ± s.d; *$P < 0.05$, **$P < 0.01$, ***$P < 0.001$. Source data are provided as a Source Data file.

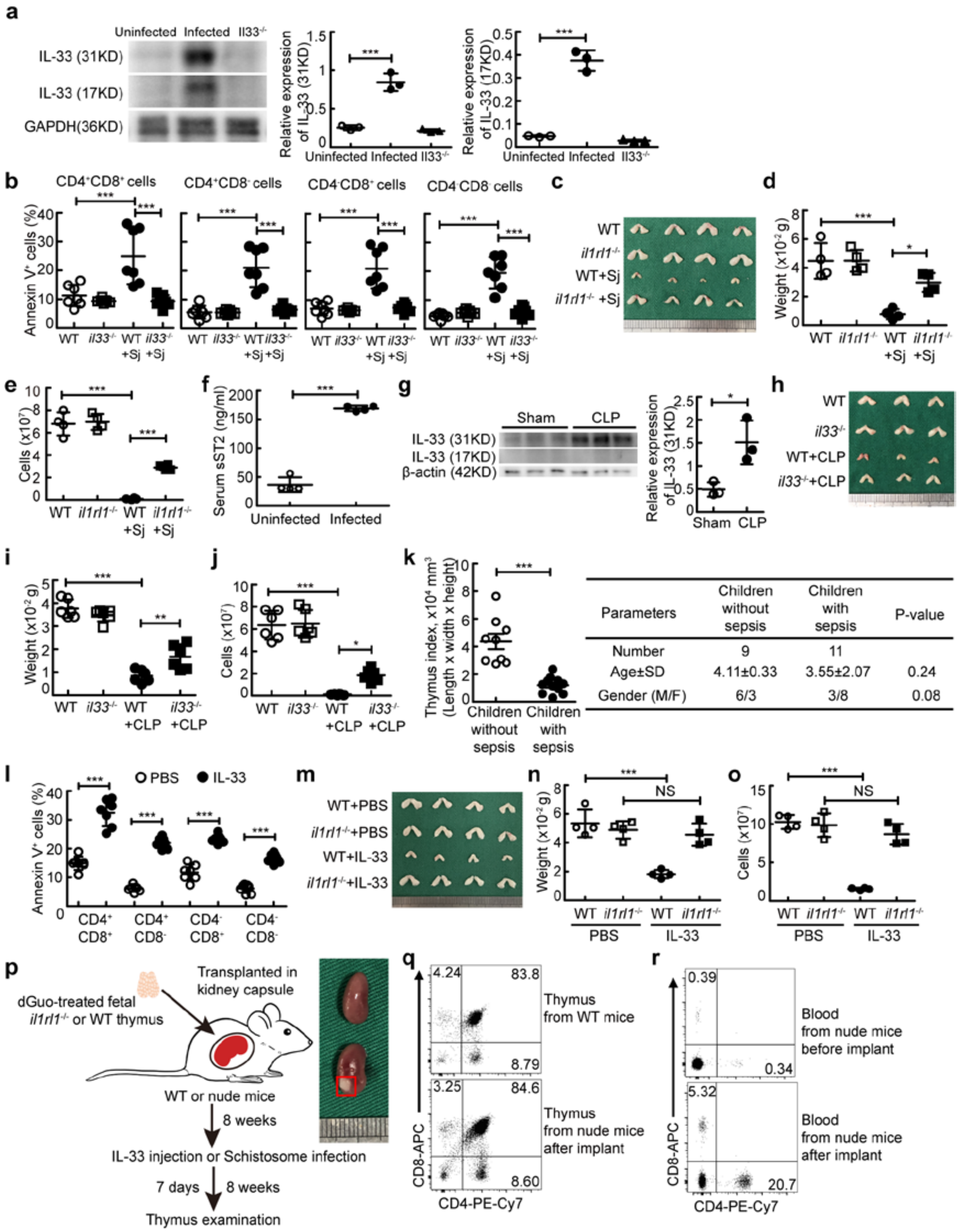

Fig. S3. IL-33 leads to thymic involution during severe infection. (**a**) Western blots of IL-33 in the liver after schistosome infection (n = 3 mice). P = 0.0001 (31KD), P < 0.0001 (17KD). (**b**) Annexin V$^+$ cells in thymocytes after schistosome infection (n = 7 mice, pool of two independent experiments). WT+Sj versus WT, P < 0.0001 for all subsets; *il33*$^{-/-}$+Sj versus WT+Sj, P < 0.0001 for all subsets. (**c-e**) Morphology, weight and cellularity of thymus from schistosome-infected mice (n = 4 mice). WT+Sj versus WT, P = 0.0002 (weight), P < 0.0001

(cells); *il33*$^{-/-}$+Sj versus WT+Sj, P = 0.0115 (weight), P = 0.0002 (cells). (**f**) The level of sST2 was determined by ELISA (n = 4 mice), P < 0.0001. (**g**) Western blots of IL-33 in the thymus after CLP induction (n = 3 mice), P = 0.0204. (**h-j**) Morphology, weight, and cellularity of thymus after CLP induction (n = 6 mice, pool of two independent experiments). WT+CLP versus WT, P < 0.0001 (weight or cells); *il33*$^{-/-}$+CLP versus WT+CLP, P = 0.0069 (weight), P = 0.0213 (cells). (**k**) Relative thymus size (n = 9 patients for children without sepsis, n = 11 patients for children with sepsis), P < 0.0001. (**l**) Annexin V^{+} cells in thymocytes after IL-33 treatment (n = 7 mice, pool of two independent experiments). P < 0.0001 for all thymocyte subsets. (**m-o**) Morphology, weight, and cellularity of thymus after IL-33 treatment (n = 4 mice). WT+IL-33 versus WT+PBS, P < 0.0001 (weight or cells); *il33*$^{-/-}$+IL-33 versus *il33*$^{-/-}$+PBS, P = 0.9063 (weight), P = 0.4730 (cells). (**p**) Morphology of the thymus in the kidney capsule after thymus transplantation. (**q**) Representative flow cytometry of thymocytes after thymus transplantation. (**r**) Representative flow cytometry of T cells in peripheral blood after thymus transplantation. (**a**, **b**, **d**, **e**, **i**, **j**, **l**, **n**, **o**) One-way ANOVA with Tukey's multiple comparisons. (**f**, **g**, **k**) Unpaired two-tailed Student’s t-test. All data are shown as the mean ± s.d. *P < 0.05, **P < 0.01, ***P < 0.001, NS, not significant. Source data are provided as a Source Data file.

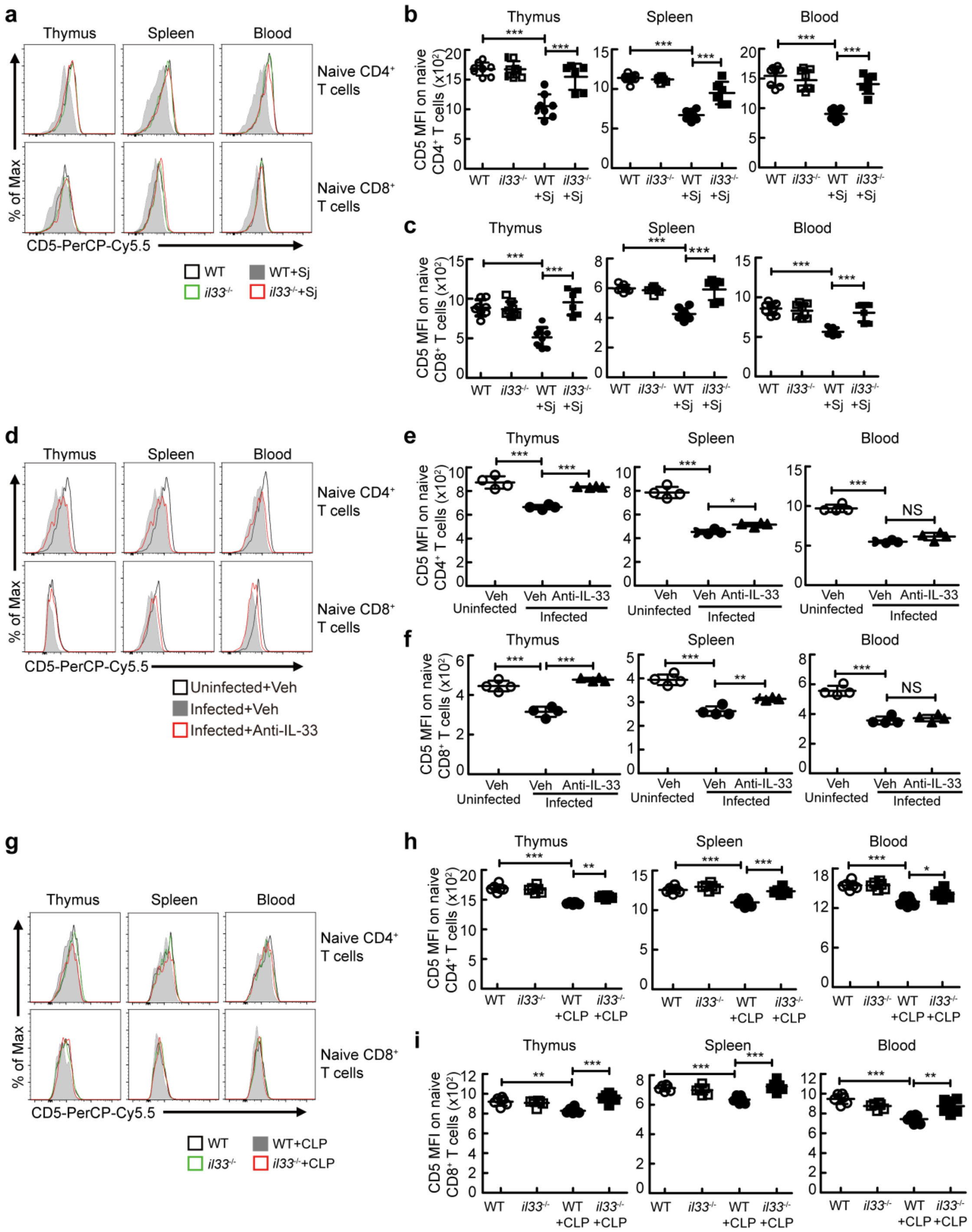

Fig. S4. Loss of IL-33-mediated thymic involution reverses T-cell immunity during severe infection. (**a-c**) Samples were from uninfected or schistosome-infected WT or *il33*$^{-/-}$ mice. CD5 MFI on naive CD4^{+} T cells or naïve CD8^{+} T cells from the thymus, spleen, or peripheral blood (*il33*$^{-/-}$+Sj, n = 6 mice, other groups, n = 8 mice, pool of two independent experiments). CD4^{+} T cells: WT+Sj versus WT, P < 0.0001 (Thymus, Spleen, or Blood); *il33*$^{-/-}$+Sj versus WT+Sj, P < 0.0001 (Thymus, Spleen, or Blood). CD8^{+} T cells: WT+Sj versus

WT, P < 0.0001 (Thymus, Spleen, or Blood); *il33*$^{-/-}$+Sj versus WT+Sj, P < 0.0001 (Thymus, Spleen, or Blood). One-way ANOVA with Tukey's multiple comparisons. (**d-f**) Samples were from uninfected or schistosome-infected mice treated with anti-IL-33. CD5 MFI on naive CD4$^+$ T or naïve CD8$^+$ T cells from the thymus, spleen, or peripheral blood (n = 4 mice). CD4$^+$ T cells: Infected+Veh versus Uninfected+Veh, P < 0.0001 (Thymus, Spleen, or Blood); Infected+anti-IL-33 versus Infected+Veh, P < 0.0001 (Thymus), P = 0.0439 (Spleen), P = 0.0899 (Blood). CD8$^+$ T cells: Infected+Veh versus Uninfected+Veh, P < 0.0001 (Thymus, Spleen, or Blood); Infected+anti-IL-33 versus Infected+Veh, P < 0.0001 (Thymus), P = 0.00559 (Spleen), P = 0.7075 (Blood). One-way ANOVA with Tukey's multiple comparisons. (**g-i**) Samples were from WT or *il33*$^{-/-}$ mice and WT or *il33*$^{-/-}$ mice after CLP induction. CD5 MFI on naive CD4$^+$ T or naïve CD8$^+$ T cells from the thymus, spleen, or peripheral blood (n = 6 mice, pool of two independent experiments). CD4$^+$ T cells: WT+CLP versus WT, P < 0.0001 (Thymus, Spleen, or Blood); *il33*$^{-/-}$+CLP versus WT+CLP, P = 0.0034 (Thymus), P < 0.0001 (Spleen), P = 0.0175 (Blood). CD8$^+$ T cells: WT+CLP versus WT, P = 0.0018 (Thymus), P = 0.0007 (Spleen), P < 0.0001 (Blood); *il33*$^{-/-}$+CLP versus WT+CLP, P < 0.0001 (Thymus), P = 0.0002 (Spleen), P = 0.0012 (Blood). One-way ANOVA with Tukey's multiple comparisons. Data are shown as the mean ± s.d. *P < 0.05, **P < 0.01, ***P < 0.001, NS, not significant. Source data are provided as a Source Data file.

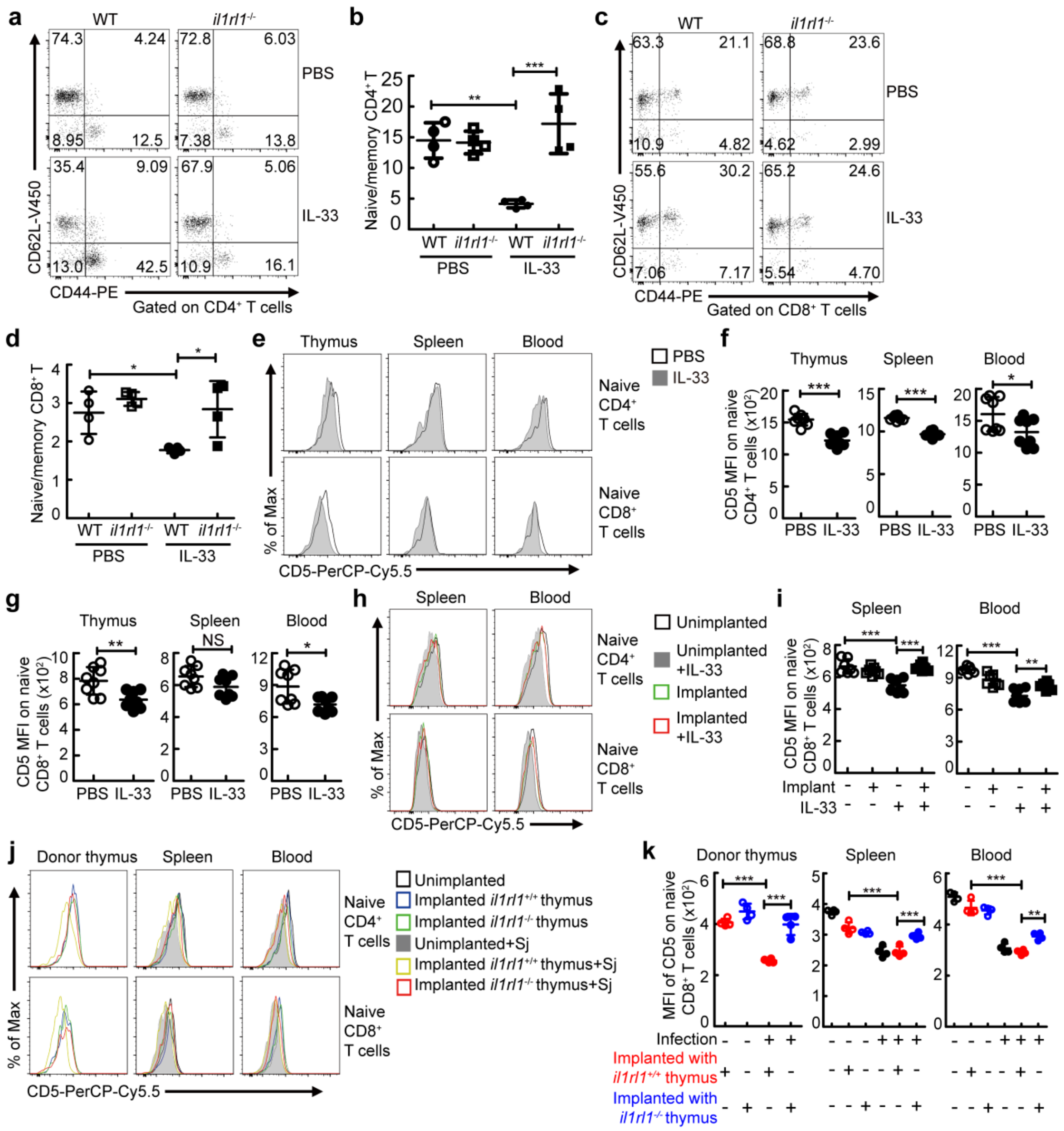

Fig. S5. IL-33 results in thymic involution-mediated T cell aging. (**a-d**) Naïve/memory naïve $CD4^+$ T cells or $CD8^+$ T cells in the spleen from WT or *il1rl1*$^{-/-}$ mice after IL-33 treatment (n = 4 mice). WT+IL-33 versus WT+PBS, P = 0.0018 ($CD4^+$ T cells), P = 0.0271 ($CD8^+$ T cells); *il1rl1*$^{-/-}$+IL-33 versus WT+IL-33, P = 0.0002 ($CD4^+$ T cells), P = 0.0237 ($CD8^+$ T cells). (**e-g**) Samples were from mice after IL-33 treatment. CD5 MFI on naive $CD4^+$ T cells or $CD8^+$ T cells from the thymus, spleen, or peripheral blood (n = 8 mice, pool of two independent experiments). $CD4^+$ T cells, P < 0.0001 (Thymus or Spleen), P = 0.0392 (Blood); $CD8^+$ T cells, P = 0.0057 (Thymus), P = 0.0676 (Spleen), P = 0.0162 (Blood); Unpaired two-tailed Student's t-test. (**h**, **i**) Mice were injected with IL-33 at week 8 after transplantation with *il1rl1*$^{-/-}$ thymus. (**h**) Representative flow cytometry of CD5 MFI on naive $CD4^+$ T cells

or $CD8^+$ T cells in the spleen or peripheral blood. (**i**) CD5 MFI on naive $CD8^+$ T cells in the spleen or peripheral blood (PBS, n = 7 mice; IL-33, n = 8 mice; pool of two independent experiments). WT+IL-33 versus WT+PBS, P < 0.0001 (Spleen), P < 0.0001 (Blood); $il1rl1^{-/-}$+IL-33 versus WT+IL-33, P < 0.0001 (Spleen), P = 0.0028 (Blood). (**j, k**) Mice were infected with schistosome at week 8 after transplantation with $il1rl1^{+/+}$ or $il1rl1^{-/-}$ thymus. (**j**) Representative flow cytometry of CD5 MFI on naive $CD4^+$ T cells or $CD8^+$ T cells in donor thymus, spleen, or peripheral blood. (**k**) CD5 MFI on naive $CD8^+$ T in donor thymus, spleen, or peripheral blood (n = 4 mice). Infected+$il1rl1^{+/+}$ thymus versus Uninfected+$il1rl1^{+/+}$ thymus, P < 0.0001 (Donor thymus, Spleen, or Blood); Infected+$il1rl1^{+/+}$ thymus versus Infected+$il1rl1^{-/-}$ thymus, P < 0.0001 (Donor thymus), P = 0.0008 (Spleen), P = 0.0017 (Blood). (**b**, **d**, **i**, **k**) One-way ANOVA with Tukey's multiple comparisons. Data are shown as the mean ± s.d. *P < 0.05, **P < 0.01, ***P < 0.001. Source data are provided as a Source Data file.

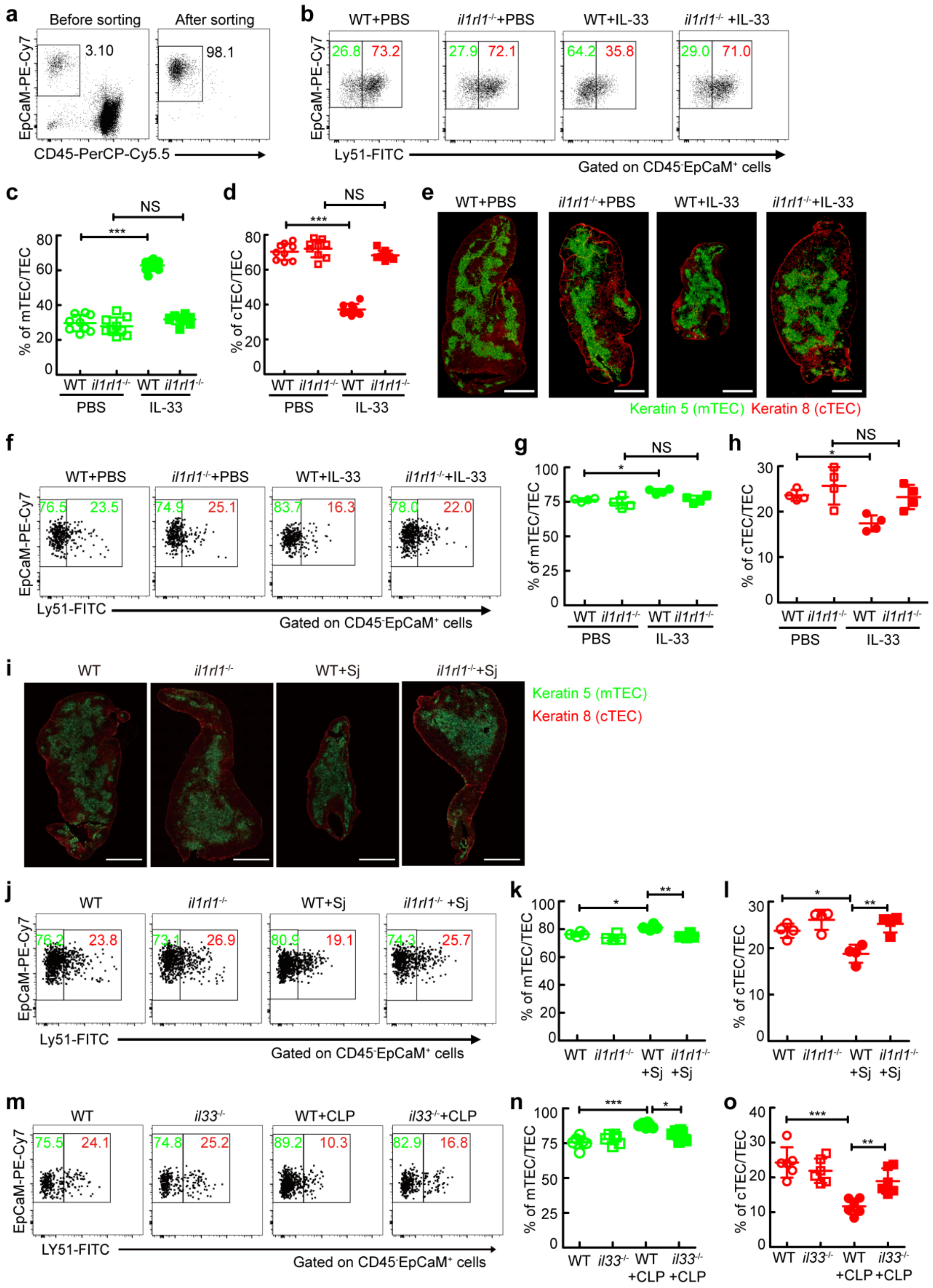

Fig. S6. IL-33 perturbs the compartment of TECs. (**a**) Representative flow cytometry of TECs before or after being sorted by MACS from the thymus cultured in FTOCs. (**b-d**) Representative and quantified flow cytometry of mTECs and cTECs in FTOC treated with

IL-33 (n = 9 biologically independent samples of fetal thymic lobes, pool of three independent experiments). WT+IL-33 versus WT+PBS, P < 0.0001 (mTEC or cTEC); *il1rl1*$^{-/-}$+IL-33 versus *il1rl1*$^{-/-}$+PBS, P = 0.2001 (mTEC), P = 0.2018 (cTEC); One-way ANOVA with Tukey's multiple comparisons. (**e**) Histology of the thymus from mice after IL-33 treatment; Green, Keratin 5; red, Keratin 8; Scale bar, 1000 μm. (**f**-**h**) Representative and quantified flow cytometry of cTECs or mTECs in WT or *il1rl1*$^{-/-}$ mice after IL-33 treatment (n = 4 mice). WT+IL-33 versus WT+PBS, P = 0.0314 (mTEC or cTEC); *il1rl1*$^{-/-}$+IL-33 versus *il1rl1*$^{-/-}$+PBS, P = 0.5786 (mTEC or cTEC); One-way ANOVA with Tukey's multiple comparisons. (**i**) Histology of the thymus from WT or *il1rl1*$^{-/-}$ mice after schistosome infection; Green, Keratin 5; red, Keratin 8; Scale bar, 1000 μm. (**j**-**l**) Representative and quantified flow cytometry of cTECs or mTECs in WT or *il1rl1*$^{-/-}$ mice after schistosome infection (n = 4 mice). WT+Infected versus WT+Uninfected, P = 0.0115 (mTEC), P = 0.0113 (cTEC); *il1rl1*$^{-/-}$+Infected versus WT+Infected, P = 0.0023 (mTEC), P = 0.0020 (cTEC); One-way ANOVA with Tukey's multiple comparisons. (**m**-**o**) Representative flow cytometry and quantification of flow cytometry of cTECs or mTECs in WT or *il33*$^{-/-}$ mice after CLP induction (n = 6 mice, pool of two independent experiments). WT+CLP versus WT, P < 0.0001 (mTEC or cTEC); *il1rl1*$^{-/-}$+CLP versus WT+CLP, P = 0.0111 (mTEC), P = 0.0094 (cTEC); One-way ANOVA with Tukey's multiple comparisons. Data are shown as the mean ± s.d. *P < 0.05, **P < 0.01, ***P < 0.001, NS, not significant. Source data are provided as a Source Data file.

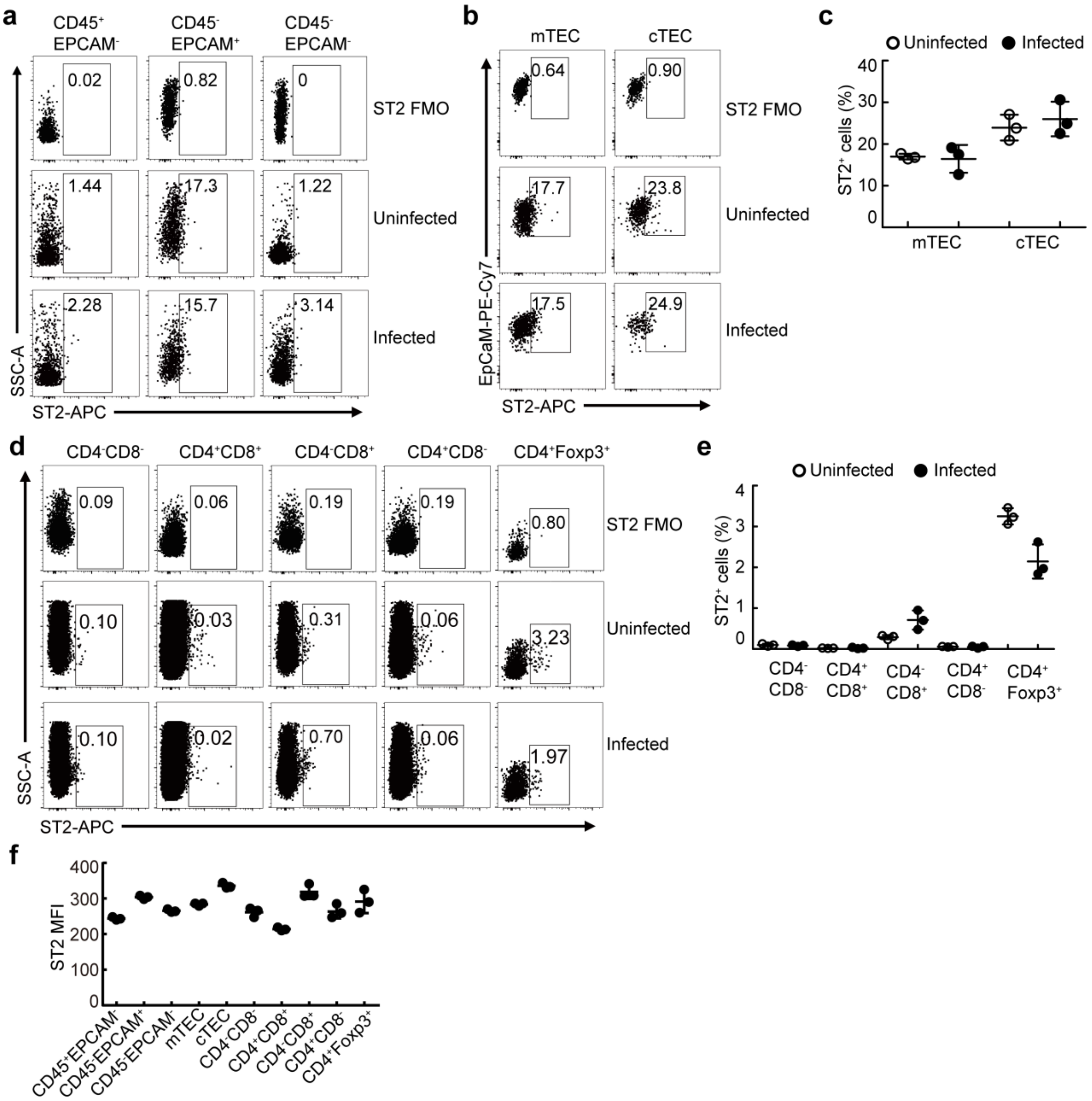

Fig. S7. The expression of ST2 on cells in the thymus. (**a-e**) Cells were from the thymus in uninfected or schistosome-infected mice. (**a**) Representative flow cytometry of ST2$^+$ cells gated on CD45$^+$EpCaM$^-$, CD45$^-$EpCaM$^+$, or CD45$^-$EpCaM$^-$ cells. (**b, c**) Representative and quantified flow cytometry of ST2$^+$ cells gated on mTECs or cTECs (n = 3 mice). (**d, e**) Representative and quantified flow cytometry of ST2$^+$ cells gated on CD4$^+$CD8$^+$DP, CD4SP, CD8SP, CD4$^-$CD8$^-$ DN, or Treg cells (n = 3 mice). (f) Quantified ST2 MFI of ST2$^+$ cells gated on CD45$^+$EpCaM$^-$, CD45$^-$EpCaM$^+$, CD45$^-$EpCaM$^-$, mTECs, cTECs, CD4$^+$CD8$^+$DP, CD4SP, CD8SP, CD4$^-$CD8$^-$ DN, or Treg cells in the thymus from schistosome-infected mice (n = 3 mice). Data are shown as the mean ± s.d. Source data are provided as a Source Data file.
