# Supplementary material for: IL-33 induces thymic involution-associated naive T cell aging and impairs host control of severe infection
Source: Nat Commun. 2022 Nov 12;13:6881. doi: 10.1038/s41467-022-34660-4 (PMC9653498; doi:10.1038/s41467-022-34660-4)
Supplement: Supplementary file 3 — Reporting Summary [file 41467_2022_34660_MOESM3_ESM.pdf]

nature portfolio

Corresponding author(s): Sha Zhou, Chuan Su, Xiaojun Chen

Last updated by author(s): Oct 26, 2022

Reporting Summary

Nature Portfolio wishes to improve the reproducibility of the work that we publish. This form provides structure for consistency and transparency in reporting. For further information on Nature Portfolio policies, see our Editorial Policies and the Editorial Policy Checklist.

Statistics

For all statistical analyses, confirm that the following items are present in the figure legend, table legend, main text, or Methods section.

n/a	Confirmed	
☐	☒	The exact sample size (*n*) for each experimental group/condition, given as a discrete number and unit of measurement
☐	☒	A statement on whether measurements were taken from distinct samples or whether the same sample was measured repeatedly
☐	☒	The statistical test(s) used AND whether they are one- or two-sided<br>*Only common tests should be described solely by name; describe more complex techniques in the Methods section.*
☒	☐	A description of all covariates tested
☐	☒	A description of any assumptions or corrections, such as tests of normality and adjustment for multiple comparisons
☐	☒	A full description of the statistical parameters including central tendency (e.g. means) or other basic estimates (e.g. regression coefficient) AND variation (e.g. standard deviation) or associated estimates of uncertainty (e.g. confidence intervals)
☐	☒	For null hypothesis testing, the test statistic (e.g. *F*, *t*, *r*) with confidence intervals, effect sizes, degrees of freedom and *P* value noted<br>*Give P values as exact values whenever suitable.*
☒	☐	For Bayesian analysis, information on the choice of priors and Markov chain Monte Carlo settings
☒	☐	For hierarchical and complex designs, identification of the appropriate level for tests and full reporting of outcomes
☒	☐	Estimates of effect sizes (e.g. Cohen's *d*, Pearson's *r*), indicating how they were calculated

Our web collection on statistics for biologists contains articles on many of the points above.

Software and code

Policy information about availability of computer code

Data collection	BD FACSuite (Version 1.0.6.5230), Pannoramic Scanner software (Version 3.0.2)
Data analysis	FlowJo (version 10.0.7) and GraphPad Prism (Version 5.0)

For manuscripts utilizing custom algorithms or software that are central to the research but not yet described in published literature, software must be made available to editors and reviewers. We strongly encourage code deposition in a community repository (e.g. GitHub). See the Nature Portfolio guidelines for submitting code & software for further information.

Data

Policy information about availability of data

All manuscripts must include a data availability statement. This statement should provide the following information, where applicable:

- Accession codes, unique identifiers, or web links for publicly available datasets
- A description of any restrictions on data availability
- For clinical datasets or third party data, please ensure that the statement adheres to our policy

The RNA-seq data of naive CD4+ T cells and TECs were deposited in the NCBI's GEO under accession number GSE189200 [https://www.ncbi.nlm.nih.gov/geo/query/acc.cgi?acc=GSE189200] and GSE189201 [https://www.ncbi.nlm.nih.gov/geo/query/acc.cgi?acc=GSE189201], respectively. All other data are included in the Article, Supplementary Information or Source Data file. Source data are provided with this paper.

Human research participants

Policy information about studies involving human research participants and Sex and Gender in Research.

Reporting on sex and gender	children without sepsis (male/female: 6/3)<br>children with sepsis (male/female: 3/8)
Population characteristics	We reviewed the chest computed tomographic (CT) images of all children with sepsis who underwent chest CT examinations (n=11) from January 2020 to November 2020 at the Children's Hospital of Nanjing Medical University. Children with funnel chest but without infectious diseases or tumors who underwent chest CT examinations served as controls (n=9). 9 males, 11 females; age from 2 to 8 years old.
Recruitment	We reviewed the chest computed tomographic (CT) images of all children with sepsis who underwent chest CT examinations from January 2020 to November 2020 at the Children's Hospital of Nanjing Medical University. Children with funnel chest but without infectious diseases or tumors who underwent chest CT examinations served as controls. No potential self-selection bias is present.
Ethics oversight	Ethical clearance for this retrospective study was obtained from the Institutional Review Board of Nanjing Medical University, Nanjing, China (Permit Number: 2020607).

Note that full information on the approval of the study protocol must also be provided in the manuscript.

Field-specific reporting

Please select the one below that is the best fit for your research. If you are not sure, read the appropriate sections before making your selection.

☒ Life sciences ☐ Behavioural & social sciences ☐ Ecological, evolutionary & environmental sciences

For a reference copy of the document with all sections, see nature.com/documents/nr-reporting-summary-flat.pdf

Life sciences study design

All studies must disclose on these points even when the disclosure is negative.

Sample size	Sample sizes were determined based on pilot studies and expert experience.
Data exclusions	No data was excluded in this study.
Replication	Most experiments were validated with repeat experiments at least 2 times. Few exceptions include costly experiments, such as anti-IL-33 neutralizing antibody treatment and RNA-sequencing, were biological replicates.
Randomization	Treatment group in mouse experiments were mixed within the same cage and groups were assigned randomly. For in vitro experiments, all cells in each experiment were randomly from mice in each group. All thymus in each experiment were from the same pool and were randomized into different treatment groups.
Blinding	Animal treatments were performed by technicians who were not blind, but not involved in sample measurement. All in vitro experiments were not blind because students have their own projects, and it is impossible for others to replace them to treat cells or thymus and analyze samples.

Reporting for specific materials, systems and methods

We require information from authors about some types of materials, experimental systems and methods used in many studies. Here, indicate whether each material, system or method listed is relevant to your study. If you are not sure if a list item applies to your research, read the appropriate section before selecting a response.

Materials & experimental systems

n/a	Involved in the study
	☒ Antibodies
☒	☐ Eukaryotic cell lines
☒	☐ Palaeontology and archaeology
	☒ Animals and other organisms
☒	☐ Clinical data
☒	☐ Dual use research of concern

Methods

n/a	Involved in the study
☒	☐ ChIP-seq
	☒ Flow cytometry
☒	☐ MRI-based neuroimaging

Antibodies

Antibodies used

The following primary antibodies were used for western blot. They are listed as antigen first, followed by supplier, catalog number, clone name, and dilution ratio.
1) IL-33, R&D Systems, AF3626, NA, 0.4 μg/mL
2) P100/P52, Cell Signaling Technology, 4882S, NA, 1:1000
3) Pou2f3, Novus Biologicals, NBP2-94551-0.1ml, NA, 1:1000
4) FoxO1, Cell Signaling Technology, 2880S, C29H4, 1:1000
5) GAPDH , Abcam, ab181602, EPR16891, 1:10000
6) β-tubulin, Cell Signaling Technology, 2146S, NA, 1:1000
7) β-actin, Cell Signaling Technology, 4970S, 13E5, 1:1000
8) HRP conjugated Anti-Goat IgG (H+L) , KPL, 5220-0362, NA, 1:1000
9) HRP conjugated Anti-mouse IgG (H+L) , Cell Signaling Technology, 7076S, NA, 1:1000
10) HRP conjugated Anti-rabbit IgG (H+L) , Cell Signaling Technology, 7074S, NA, 1:1000

The following primary antibodies were used for flow cytometry. They are listed as antigen first, followed by supplier, catalog number, clone name and dilution ratio.
1) CD16/32, Invitrogen, 14-0161-86, 93, 1:100
2) FITC anti-mouse CD3e, Invitrogen, 11-0031-82, 145-2C11, 1:400
3) V450 anti-Mouse CD3e, bdbiosciences, 560804, 500A2, 1:500
4) PE-Cyanine7 anti-mouse CD4, Invitrogen, 25-0042-82, RM4-5, 1:500
5) FITC anti-mouse CD4, Invitrogen, 11-0042-85, RM4-5, 1:400
6) PE-Cyanine7 anti-mouse CD25, Invitrogen, 25-0251-82, PC61.5, 1:400
7) PE anti-mouse FOXP3, Invitrogen, 12-5773-82, FJK-16s, 1:40
8) APC anti-mouse CD8a, Invitrogen, 17-0081-82, 53-6.7, 1:1000
9) FITC anti-mouse CD8a, Invitrogen, 11-0081-82, 53-6.7, 1:200
10) PE anti-mouse CD44, Invitrogen, 12-0441-82, IM7, 1:500
11) BV421 anti-Mouse CD62L, bdbiosciences, 562910, MEL-14, 1:400
12) PerCP-Cyanine5.5 anti-Mouse CD5, Invitrogen, 45-0051-80, 53-7.3, 1:1000
13) APC anti-mouse Annexin V Recombinant Protein, Invitrogen, BMS306APC-100, NA, 1:100
14) Alexa Fluor 647 anti-mouse Ki-67, bdbiosciences, 558615, B56, 1:100
15) FITC anti-mouseCD249 (Ly51), Invitrogen, 11-5891-82, 6C3, 1:500
16) PerCP-Cyanine5.5 anti-Mouse CD45, Invitrogen, 45-0451-82, 30-F11, 1:1000
17) PE-Cyanine7 anti-mouse CD326 (EpCAM), Invitrogen, 25-5791-80, G8.8, 1:1000
18) APC anti-mouse IL-33R (ST2), Invitrogen, 17-9335-82, RMST2-2, 1:150
19) Brilliant Violet 421anti-mouse I-A/I-E (MHC class II), BioLegend, 107632, M5/114.15.2, 1:1000
20) FITC anti-mouse CD104 (ITGB4), BioLegend, 123606, 346-11A, 1:100
21) Alexa Fluor 647 anti-mouse L1CAM, R&D Systems, FAB5674R-100UG, NA, 1:40
22) PE anti-mouse Ly-6D, Invitrogen, 12-5974-80, 49-H4, 1:1000

The following primary antibodies were used for immunofluorescence. They are listed as antigen first, followed by supplier, catalog number, clone name and dilution ratio.
1) Anti-Cytokeratin 5, Abcam, ab52635, EP1601Y, 1:100
2) Anti-Cytokeratin 8, DSHB, TROMA-I, NA, 1:10
3) Alexa Fluor 555 Conjugate Anti-rat IgG (H+L), Cell Signaling Technology, 4417S, 1:500
4) Alexa Fluor 488 Conjugate Anti-rabbit IgG (H+L) , Cell Signaling Technology, 4412S, 1:500

The following primary antibodies were used for in vitro/in vivo treatment. They are listed as antigen first, followed by supplier, catalog number, clone name and dilution ratio.
1) Mouse IL-33 Antibody, R&D, AF3626, NA, 3.6 μg /20g
2) Purified anti-mouse CD3 Antibody, BioLegend, 100238, 17A2, 0.1μg/100μl
3) Purified anti-mouse CD28 Antibody, BioLegend, 102116, 37.51, 0.3μg/100μl

Validation

All antibodies were commercially available, and validated by manufacturers and/or citations. Manufacturer websites containing their validation data and/or citations, are listed below:
WB antibodies:
1) IL-33 (R&D Systems, AF3626): website (https://www.rndsystems.com/cn/products/mouse-il-33-antibody_af3626)
2) P100/P52 (Cell Signaling Technology, 4882S): website (https://www.cellsignal.cn/products/primary-antibodies/nf-kb2-p100-p52-antibody/4882?site-search type=Products&N=4294956287&Ntt=4882&fromPage=plp)
3) Pou2f3 (Novus Biologicals, NBP2-94551-0.1ml): website (https://www.novusbio.com/products/pou2f3-antibody_nbp2-94551)

4) FoxO1 (Cell Signaling Technology, 2880S): website (https://www.cellsignal.cn/products/primary-antibodies/foxo1-c29h4-rabbit-mab/2880?site-search-type=Products&N=4294956287&Ntt=2880&fromPage=plp&_requestid=5148298)
5) GAPDH(Abcam, ab181602) : website (https://www.abcam.cn/gapdh-antibody-epr16891-loading-control-ab181602.html)
6) β-tubulin (Cell Signaling Technology, 2146S): website (https://www.cellsignal.cn/products/primary-antibodies/b-tubulin-antibody/2146?site-search-type=Products&N=4294956287&Ntt=2146&fromPage=plp)
7) β-actin (Cell Signaling Technology, 4970S): website (https://www.cellsignal.cn/products/primary-antibodies/b-actin-13e5-rabbit-mab/4970?site-search-type=Products&N=4294956287&Ntt=4970&fromPage=plp&_requestid=5149225)
8) HRP conjugated Anti-Goat IgG (H+L) (KPL, 5220-0362) : website (https://seracare.com/AntiGoat-IgG-HL-Antibody-PeroxidaseLabeled-5220-0362/)
9) HRP conjugated Anti-mouse IgG (H+L) (Cell Signaling Technology, 7076S) : website (https://www.cellsignal.cn/products/secondary-antibodies/anti-mouse-igg-hrp-linked-antibody/7076?site-search-type=Products&N=4294956287&Ntt=7076s&fromPage=plp&_requestid=5123243)
10) HRP conjugated Anti-rabbit IgG (H+L) (Cell Signaling Technology, 7074S): website (https://www.cellsignal.cn/products/secondary-antibodies/anti-rabbit-igg-hrp-linked-antibody/7074?site-search-type=Products&N=4294956287&Ntt=7074s&fromPage=plp&_requestid=5184395)

Flow cytometry antibodies
1) CD16/32 (Invitrogen, 14-0161-86) : website （https://www.thermofisher.cn/cn/zh/antibody/product/CD16-CD32-Antibody-clone-93-Monoclonal/14-0161-86）
2) FITC anti-mouse CD3e (Invitrogen, 11-0031-82) : website (https://www.thermofisher.cn/cn/zh/antibody/product/CD3e-Antibody-clone-145-2C11-Monoclonal/11-0031-82)
3) V450 anti-Mouse CD3e (bdbiosciences, 560804) : website （https://www.bdbiosciences.com/en-us/products/reagents/flow-cytometry-reagents/research-reagents/single-color-antibodies-ruo/v450-hamster-anti-mouse-cd3e.560804）
4) PE-Cyanine7 anti-mouse CD4 (Invitrogen, 25-0042-82) : website （https://www.thermofisher.cn/cn/zh/antibody/product/CD4-Antibody-clone-RM4-5-Monoclonal/25-0042-82）
5) FITC anti-mouse CD4 (Invitrogen, 11-0042-85) : website （https://www.thermofisher.cn/cn/zh/antibody/product/CD4-Antibody-clone-RM4-5-Monoclonal/11-0042-85）
6) PE-Cyanine7 anti-mouse CD25 (Invitrogen, 25-0251-82) : website （https://www.thermofisher.cn/cn/zh/antibody/product/CD25-Antibody-clone-PC61-5-Monoclonal/25-0251-82）
7) PE anti-mouse FOXP3 (Invitrogen, 12-5773-82) : website （https://www.thermofisher.cn/cn/zh/antibody/product/FOXP3-Antibody-clone-FJK-16s-Monoclonal/12-5773-82）
8) APC anti-mouse CD8a (Invitrogen, 17-0081-82) : website （https://www.thermofisher.cn/cn/zh/antibody/product/CD8a-Antibody-clone-53-6-7-Monoclonal/17-0081-82）
9) FITC anti-mouse CD8a (Invitrogen, 11-0081-82) : website （https://www.thermofisher.cn/cn/zh/antibody/product/CD8a-Antibody-clone-53-6-7-Monoclonal/11-0081-82）
10) PE anti-mouse CD44 (Invitrogen, 12-0441-82) : website （https://www.thermofisher.cn/cn/zh/antibody/product/CD44-Antibody-clone-IM7-Monoclonal/12-0441-82）
11) BV421 anti-Mouse CD62L (bdbiosciences, 562910) : website （https://www.bdbiosciences.com/en-us/products/reagents/flow-cytometry-reagents/research-reagents/single-color-antibodies-ruo/bv421-rat-anti-mouse-cd62l.562910）
12) PerCP-Cyanine5.5 anti-Mouse CD5 (Invitrogen, 45-0051-80) : website （https://www.thermofisher.cn/cn/zh/antibody/product/CD5-Antibody-clone-53-7-3-Monoclonal/45-0051-80）
13) APC anti-mouse Annexin V Recombinant Protein(Invitrogen, BMS306APC-100) : website （https://www.thermofisher.cn/cn/zh/antibody/product/Annexin-V-Recombinant-Protein/BMS306APC-100）
14) Alexa Fluor 647 anti-mouse Ki-67 (bdbiosciences, 558615) : website(https://www.bdbiosciences.com/en-us/products/reagents/flow-cytometry-reagents/research-reagents/single-color-antibodies-ruo/alexa-fluor-647-mouse-anti-ki-67.558615)
15) FITC anti-mouseCD249 (Ly51) (Invitrogen, 11-5891-82) : website （https://www.thermofisher.cn/cn/zh/antibody/product/CD249-BP-1-Antibody-clone-6C3-Monoclonal/11-5891-82）
16) PerCP-Cyanine5.5 anti-Mouse CD45 (Invitrogen, 45-0451-82) : website （https://www.thermofisher.cn/cn/zh/antibody/product/CD45-Antibody-clone-30-F11-Monoclonal/45-0451-82）
17) PE-Cyanine7 anti-mouse CD326 (EpCAM) (Invitrogen, 25-5791-80) : website (https://www.thermofisher.cn/cn/zh/antibody/product/CD326-EpCAM-Antibody-clone-G8-8-Monoclonal/25-5791-80)
18) APC anti-mouse IL-33R (ST2) (Invitrogen, 17-9335-82) : website (https://www.thermofisher.cn/cn/zh/antibody/product/IL-33R-ST2-Antibody-clone-RMST2-2-Monoclonal/17-9335-82)
19) Brilliant Violet 421anti-mouse I-A/I-E (MHC class II) (BioLegend, 107632) : website(https://www.biolegend.com/en-us/products/brilliant-violet-421-anti-mouse-i-a-i-e-antibody-7147)
20) FITC anti-mouse CD104 (ITGB4) (BioLegend, 123606) : website(https://www.biolegend.com/en-us/products/fitc-anti-mouse-cd104-antibody-4491)
21) Alexa Fluor 647 anti-mouse L1CAM (R&D Systems, FAB5674R-100UG) : website(https://www.rndsystems.com/cn/products/mouse-l1cam-alexa-fluor-647-conjugated-antibody-555_fab5674r)
22) PE anti-mouse Ly-6D(Invitrogen, 12-5974-80) : website (https://www.thermofisher.cn/cn/zh/antibody/product/Ly-6D-Antibody-clone-49-H4-Monoclonal/12-5974-80)

Immunofluorescence antibodies:
1) Anti-Cytokeratin 5 (Abcam, ab52635): website(https://www.abcam.cn/cytokeratin-5-antibody-ep1601y-cytoskeleton-marker-ab52635.html)
2) Anti-Cytokeratin 8 (DSHB, TROMA-I): website (https://dshb.biology.uiowa.edu/TROMA-I)
3) Alexa Fluor 555 Conjugate Anti-rat IgG (H+L) (Cell Signaling Technology, 4417S): website(https://www.cellsignal.cn/products/secondary-antibodies/anti-rat-igg-h-l-alexa-fluor-555-conjugate/4417?site-search-type=Products&N=4294956287&Ntt=4417s&fromPage=plp&_requestid=5206265)
4) Alexa Fluor 488 Conjugate Anti-rabbit IgG (H+L) (Cell Signaling Technology, 4412S): website(https://www.cellsignal.cn/products/secondary-antibodies/anti-rabbit-igg-h-l-f-ab-2-fragment-alexa-fluor-488-conjugate/4412?site-search-type=Products&N=4294956287&Ntt=4412s&fromPage=plp&_requestid=5206887)

Vitro/vivo treatment antibodies:

1) Mouse IL-33 Antibody (R&D, AF3626): website (https://www.rndsystems.com/cn/products/mouse-il-33-antibody_af3626)
2) Purified anti-mouse CD3 Antibody (BioLegend, 100238): website (https://www.biolegend.com/en-us/products/ultra-leaf-purified-anti-mouse-cd3-antibody-8078)
3) Purified anti-mouse CD28 Antibody (BioLegend, 102116): website (https://www.biolegend.com/en-us/products/ultra-leaf-purified-anti-mouse-cd28-antibody-7733)

Animals and other research organisms

Policy information about studies involving animals; ARRIVE guidelines recommended for reporting animal research, and Sex and Gender in Research

Laboratory animals	Six to eight week-old Wild-type C57BL/6J mice and nude mice were purchased from the Animal Core Facility of Nanjing Medical University (Nanjing, China). IL-33-deficient (il-33–/–) C57BL/6J mice with replacement of exon 2 with a tandemly arrayed promoter-less GFP gene and a floxed neomycin resistance gene and ST2-deficient (il1rl1–/–) C57BL/6J mice with deletion of exon 3 were obtained from Dr. Hong Zhou (Anhui Medical University, Hefei, China). Pou2f3-/- mice with deletion of exon 3 were obtained from Dr. Minjun Ji (Nanjing Medical University, Nanjing, China). All mice were housed under 20-22℃ with a 12-hour light/dark cycle and specific pathogen-free with humidity between 40% and 60% in the Animal Core Facility of Nanjing Medical University.
Wild animals	The study did not involve wild animals.
Reporting on sex	We used age-matched male mice for all of the experiments.
Field-collected samples	The study did not involve samples collected from the field.
Ethics oversight	All animal procedures were conducted following the Regulations for the Administration of Affairs Concerning Experimental Animals (1988.11.14) and approved by the Institutional Animal Care and Use Committee (IACUC) for the use of laboratory animals at Nanjing Medical University (Permit Number: IACUC-1712022).

Note that full information on the approval of the study protocol must also be provided in the manuscript.

Flow Cytometry

Plots

Confirm that:

☒ The axis labels state the marker and fluorochrome used (e.g. CD4-FITC).

☒ The axis scales are clearly visible. Include numbers along axes only for bottom left plot of group (a 'group' is an analysis of identical markers).

☒ All plots are contour plots with outliers or pseudocolor plots.

☒ A numerical value for number of cells or percentage (with statistics) is provided.

Methodology

Sample preparation	At the indicated time points, single-cell suspensions were prepared from the spleen, thymus, or peripheral blood of individual animals. Single-cell suspensions were blocked with anti-mouse CD16/32 (Thermo Fisher Scientific) before being stained with the antibodies.
Instrument	BD FACSVerse
Software	BD FACSuite (Version 1.0.6.5230)
Cell population abundance	For flow cytometry, 500ul murine blood, 2 million splenocytes or thymocytes were stained.
Gating strategy	1. FSC and SSC gates were used to select for single cells.<br>2. mTEC subsets, the gating strategy is provided in figure 7D.<br>3. CD5 expression on naive T cells, the gating strategy is provided in figure S1A.<br>4. CD3+CD4+CD8-CD44-CD62L+gate strategy was used to identify naive CD4+T cells.<br>5. CD3+CD4-CD8+CD44-CD62L+gate strategy was used to identify naive CD8+T cells.<br>6. CD45-EPcam+gate strategy was used to identify TEC cells.<br>7. CD45-EPcam+Ly51+gate strategy was used to identify cTEC cells.<br>8. CD45-EPcam+Ly51-gate strategy was used to identify mTEC cells.<br>9. CD45-EPcam+MHCII-ITGB4+L1cam-gate strategy was used to identify mTECI cells.<br>10. CD45-EPcam+MHCII+ly6D-gate strategy was used to identify mTECII cells.<br>11. CD45-EPcam+MHCII+ly6D+ and CD45-EPcam+MHCII-ITGB4-L1cam-ly6D+ gate strategy was used to identify mTECIII cells.<br>12. CD45-EPcam+MHCII-L1cam+gate strategy was used to identify mTECIV cells.<br>13. CD45+CD3+CD4+CD25+Foxp3+gate strategy was used to identify Treg cells.

☒ Tick this box to confirm that a figure exemplifying the gating strategy is provided in the Supplementary Information.
